# Flat Focusing Mirror

**DOI:** 10.1038/srep06326

**Published:** 2014-09-17

**Authors:** Y. C. Cheng, S. Kicas, J. Trull, M. Peckus, C. Cojocaru, R. Vilaseca, R. Drazdys, K. Staliunas

**Affiliations:** 1 Departament de Física i Enginyeria Nuclear, Universitat Politècnica de Catalunya, Rambla Sant Nebridi 22, 08222 Terrassa, Barcelona Spain; 2 State Research Institute for Physical Sciences and Technology, Savanorių pr. 231, 02300 Vilnius, Lithuania; 3 Laser Research Center, Department of Quantum Electronics, Vilnius University, Saulėtekio Ave. 10, LT-10222 Vilnius, Lithuania; 4 Institució Catalana de Recerca i Estudis Avançats (ICREA), Pg. Lluis Companys, 23, 08010, Barcelona, Spain

## Abstract

The control of spatial propagation properties of narrow light beams such as divergence, focusing or imaging are main objectives in optics and photonics. In this letter, we propose and demonstrate experimentally a flat focusing mirror, based on an especially designed dielectric structure without any optical axis. More generally, it also enables imaging any light pattern in reflection. The flat focusing mirror with a transversal invariance can largely increase the applicability of structured photonic materials for light beam propagation control in small-dimension photonic circuits.

It is known that spatially micro-modulated materials, such as photonic crystals (PhCs), can modify the flow of light inside and behind them both in frequency and spatial domain^1^,^2^. Particularly, beam spatial dispersion (diffractive broadening) can be manipulated, reduced or even made negative (anomalous), leading to the concept of flat lens^3^,^4^ in thin PhCs slices^5^,^6^,^7^ or meta-materials^8^,^9^,^10^, or even in acoustics, so called sonic crystals^11^, with the results of beam focusing or pattern imaging.

The principle of flat lensing is based on an anomalous angular dispersion: the plane-wave components of a beam propagating in a given material at larger angles with respect to the optical axis experience smaller phase delays. The focusing, or generally the imaging, occurs at a distance behind the flat lens where the anomalous angular dispersion gained inside the material is exactly compensated by the normal dispersion behind it (in vacuum, air, or any normal dispersion material). In this way, for a flat lens of focal distance *f* the following relation holds: *f* = *l_object_* + *l_image_*, which involves the distances to the object and to the image (note the difference with the well-known paraxial formula for spherical lenses 1/*f* = 1/*l_object_* + 1/*l_image_*).

The proposed concept of ***flat focusing mirror*** relies also on the angle-dependent anomalous phase delays of plane-wave components, which now occur during reflection and not in transmission. The expected behavior of the reflected beam is impossible for conventional metallic or dielectric Bragg mirrors where the waves reflect directly or almost directly from the mirror interface. The phase delays can be manipulated if the reflecting wave penetrates substantially into the structure and, specially, if the reflection depth depends on the incidence angle of the plane wave in a particular way.

We note that the far field focusing in reflections from flat surfaces has been recently demonstrated: in reflections from flat plasmonic metasurfaces^12^ or from high-index-contrast sub-wavelength gratings^13^,^14^. The flat surfaces in the latter studies^12^,^13^,^14^ are, however, not laterally invariant: the local modulation of the periods, or of the size of sub-wavelength structures vary across the structures, thus imposing the parabolic phase profile of reflecting wave. Such focusing is the far field effect, where the physical principles of wave propagation are similar to that in normal (Fresnel-) lenses, or in spherical mirrors. Here we propose laterally invariant near-field focusing.

In principle, chirped dielectric mirrors can provide the angle-dependent anomalous phase delays of plane-wave components, and consequently, can provide a manipulation of the angular dispersion^15^. Chirped dielectric mirrors are nowadays used to modify a ***chromatic*** dispersion, and thus to compensate dispersive broadening of ultra-short pulses^16^,^17^. At normal incidence the reflection of the different frequency components takes place at different depths due to the photonic band-gap central-frequency (Bragg frequency) variation along the chirped structure. The same principle should work considering the ***incidence angle*** instead of frequency: the plane-wave components of a monochromatic beam can reflect at different depths depending on their incidence angle, as illustrated in Fig. 1a. In case of normal dispersion, the lateral shift is directly proportional to the incidence angle, as indicated by blue dashed lines in Fig. 1c. On the other hand, the anomalously angle-dependent phase delays (depicted by the green color in Fig. 1c) could thereby result in beam focusing, as illustrated in Fig. 1b.

Nontrivial angular phase delay in reflection from a linearly chirped mirror has indeed recently been shown^15^, although a substantial focusing of the reflected beams was not obtained. This is because the anomalous angular dispersion of the linearly chirped mirrors was weak in magnitude and, moreover, it is not of a parabolic shape, which is a necessary condition for aberration-free focusing. In this work, we have adopted a new approach: we have designed and built a particular dielectric mirror structure, which provides the predefined angular delays needed for strong focusing, and then we have demonstrated such focusing by numerical simulations and experiments.

In order to design a mirror with the desired angular dependence of phase delay, we consider two important magnitudes characterizing beam propagation: beam's lateral shift *s* = −*dφ*/*dk_x_* (*φ* is phase delay and *k_x_* is the component of wave-vector in transverse *x* direction) and a magnitude proportional to the second derivative of the phase, 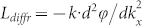,which has the sense of an equivalent diffractive propagation distance in vacuum. Note that these two quantities are related: *L_diffr_* = *k*·*ds*/*dk_x_*. The focal distance of the mirror is thus *f* = −*L_diffr_*, according to the discussion above on the principle of flat lensing. We note that the flat lensing relation *f* = *l_object_* + *l_image_* also holds for reflection from the flat mirror.

For focusing, *f* > 0, the angular dispersion has to be anomalous *L_diffr_* < 0; i.e., the plane wave components incident at larger angle should experience smaller phase delay, thus shorter penetration depth. For strong focusing, larger negative diffraction distances *L_diffr_* or, equivalently, larger negative slopes of the lateral shift *s* as a function of the incidence angle should be realized in order to obtain a maximally broad range of the incidence angles.

To design a structure with these properties we defined a target function s(*k_x_*) or, equivalently, *s(α)*, where *α* is the plane-wave incidence angle, such as the red-dashed line in Fig. 2b. A numerical iteration technique was applied to design a multi-layer mirror structure with the predefined dispersion by combining a “needle optimization” technique^18^ and a gradual evolution approach^19^. The optimization procedure converged to a specific design structure, consisting in our particular case of 98 layers, which provides a segment of linear angular dispersion of around 8 degrees, as depicted in Fig. 2b. For simplicity, the structure with a defined dispersion was designed for TE polarization only, the dispersion for TM polarization being “uncontrolled” (advanced optimization techniques in principle could allow tailoring dispersions for both polarizations simultaneously). Interestingly the optimized mirror, as depicted in Fig. 2a, can be identified as a combination between a chirped mirror structure and a GTI structure (Gires-Tournois Interfereometer^20^). Whereas chirped mirrors provide weak dispersion in a broad spectral range, a GTI structure can provide larger dispersion in a narrow spectral range^21^. Note that the mirror structure providing the required angular dispersion was not predefined by any physical arguments. It “uncontrollably” evolved during the iterations of numerical optimization procedure starting from different initial designs.

Intensity distributions of the reflected beam were calculated using the transfer matrix method. Fig. 2c shows the focusing effect at the particular incidence angle *α* = 46° corresponding to the middle of the area with negative slope of the angular dispersion curve. The incident beam was focused at a distance of 100 μm in front of the mirror surface with a beam waist diameter 2w_o_ = 8 μm. This corresponds to a Rayleigh range of 94 μm for *λ* = 532 nm, and 80 mrad total divergence angle. A clear focalization in reflection, with a minimum beam width of 8.5 μm, was obtained with maximum power concentration at 150 μm in front of the mirror along the beam axis direction, for TE polarized beam. This results in a focal distance of 250 μm. Since the focusing effect occurs only within the incidence plane, the beam profiles for TE polarization show elliptic shapes with the shorter axis in the direction of incidence plane, as depicted in Fig. 2c. The dependence of the reflected beam profile on the incidence angle is shown as a movie-1 in supplementary material which shows focusing in the angular range of around 5 degrees (from 45 to 49 degrees).

Mirror samples for experimental measurements were fabricated using ion beam sputtering technique^22^ and Ta_2_O_5_ and SiO_2_ were used as high and low refractive index materials, respectively. The experimental setup implemented to observe the focusing effect in reflection is shown in Fig. 3a. The incident beam at 532 nm, from a CW solid state laser, was focused by a x20 microscope objective at a distance of 40 μm in front of the mirror (using a high resolution closed loop step motor), with a diameter of 8 μm. The reflected beam, imaged through a long-working distance infinitely corrected x50 microscope objective and a *f* = 20 cm tube lens, was recorded by a CCD camera. The position of the x50 imaging objective and consequently the plane imaged by the CCD camera was precisely controlled with a high resolution (0.5 μm) closed-loop step motor.

We observe that the cross-sections of the reflected beam are strongly elongated ellipses, i.e. the beam width in the incidence plane (along the *x* axis) is substantially smaller than along the perpendicular direction (*y* axis). Fig. 3b shows the measured reflected beam profiles in *x* direction, obtained from the recorded CCD images, at different propagation distances, in good correspondence with the predicted behavior in Fig. 2c. The beam profiles in *x* direction, for propagation distances ranging from 0 to 240 μm, recorded in steps of 5 μm, are shown in Fig. 3c for both polarizations, showing the strongest focusing at *l*_image_ = 110 μm*,* in the TE case, for α = 46°. The focal length is thus estimated to be *f* = *l_image_ + l_object_ = * 150 μm*,* checked to remain constant for varying *l_object_*. For comparison, the focusing peak is absent for TM polarized beam measurements, which corresponds well to the numerical simulations. The numerically calculated focal distance *f* *= * 40 μm for TM polarized beam is too short to be observed, since *l*_object_ = 40 μm*.* The cross sections of the field distributions at different distances along the reflected beam at the optimum incidence angle of 46° are presented as movie-2 of supplementary materials.

The experimentally measured focal length for TE polarized beam, *f = * 150 μm, is smaller than the numerically estimated one, *f = * 250 μm, for the same structure. The discrepancy is due to fabrication errors, which for the dielectric mirror of 98 layers is hardly possible to avoid. To be confident on the role of fabrication errors, we performed a numerical simulation introducing a random error in the thickness of the layers. The randomization of the width of the layers by 1.5% (which is the estimated fabrication precision of our samples) results in reduction of the focal distance by around 50–100 μm*.* The focusing was experimentally obtained in the range of incidence angles from 45 to 47 degrees, in a smaller than that calculated numerically. This difference can be also attributed so the fabrication errors of the thickness of the layers. The maximal focal length is obtained at the incidence angle 46 degrees in a good agreement with our simulation result.

The reported flat focusing mirror contains some apparent limitations: focalization appears only at nonzero incidence angles, and it occurs only in one lateral direction. The latter limitation can be resolved for instance by considering 2D mirror geometries, as e.g., a 90 degree two-mirror corner as shown in Fig. 4a. The insets show that the beam would be tightly focused and would keep cylindrical symmetry after the two complementary reflections, as calculated numerically.

Concerning possible applications, we propose a scheme for a planar waveguide in Fig. 4b: Numerical calculations of beam propagation in a Fabry-Perot resonator composed of two such mirrors show that the beam remains tightly collimated after multiple reflections from the walls, due to focusing in reflections. This proposal takes advantage from the result that the focusing occurs at nonzero angle. Finally, Fig. 4c proves that the proposed flat focusing mirror not only focuses beams but can also image arbitrary small scale light patterns. Here a specific example has been numerically calculated, where a simple field pattern consisting of two narrow beams is restored after reflection.

In conclusion, we have proposed, and experimentally demonstrated, a new concept of flat focusing mirror or, generally, flat imaging mirror without any optical axis. This mirror can focus and reproduce transverse light patterns benefiting, at the same time, from lateral translational invariance. The focusing distances are in the micrometer range, reaching typical distances of 150–250 μm, which make the mirror suitable to build small-scale photonic devices or circuits, in which narrow beams could propagate diffraction-free without a waveguide.

## Author Contributions

**Figure 1:**
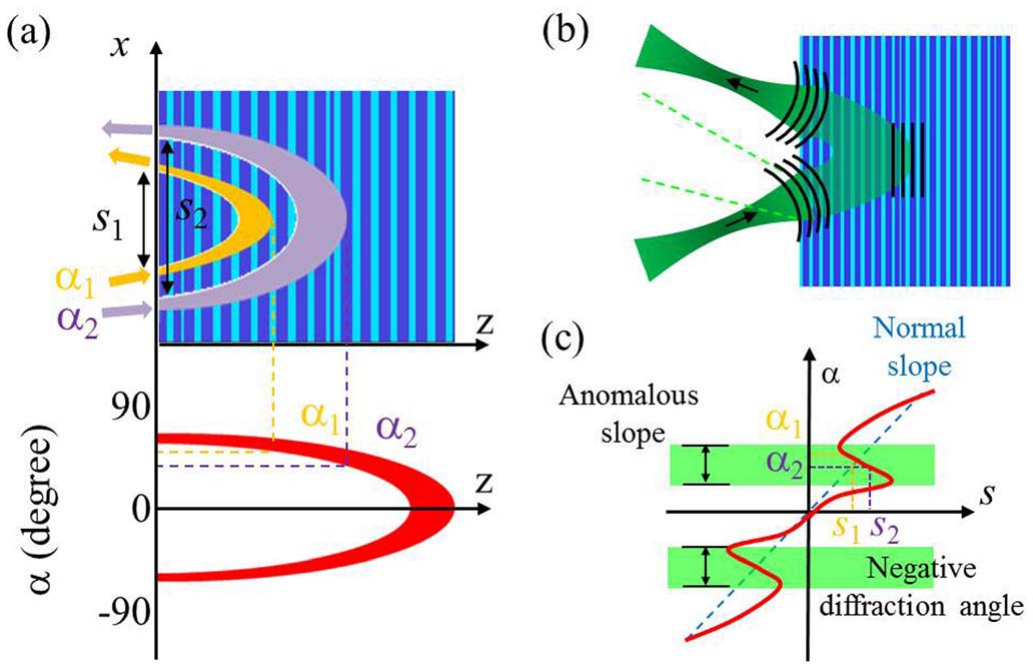
Principle of a flat focusing mirror. (a) Plane wave components incident at different angles reflect at different depths inside the structure and thus experience different phase delays and lateral shifts s. (b) Illustration of a beam focusing in reflection with flat mirror without any optical axis. The green dashed lines indicate the width of the reference beam (reflecting directly from the surface of mirror). The dark lines indicate the wave fronts at three particular positions. (c) Focusing appears at the negative diffraction angles when the lateral shift is inversely proportional to the incidence angle (anomalous slope) as shown in the green color.

**Figure 2:**
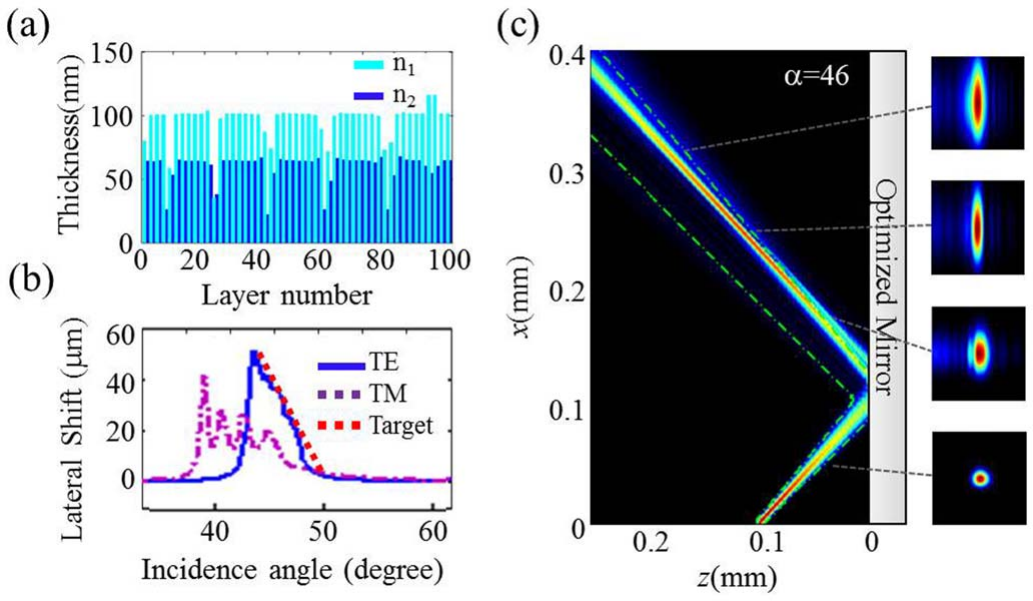
The mirror with optimized structure. (a) Physical thickness of the layers in the structure consisting of alternating layers of low n_1_ = 1.54 and high n_2_ = 2.17 refractive index material. (b) Derivative of the spatial dispersion, proportional to the lateral shift of the wave, versus incidence angle. The target function is depicted in red dashed line. (c) Beam profile at the optimum angle α = 46° for TE polarization, and the resulting 2D cross section of the reflected beam at different propagation distances (see movie-1 in supplementary material for field profile for varying incidence angle). Green dashed line represents the reflection from a conventional mirror.

**Figure 3:**
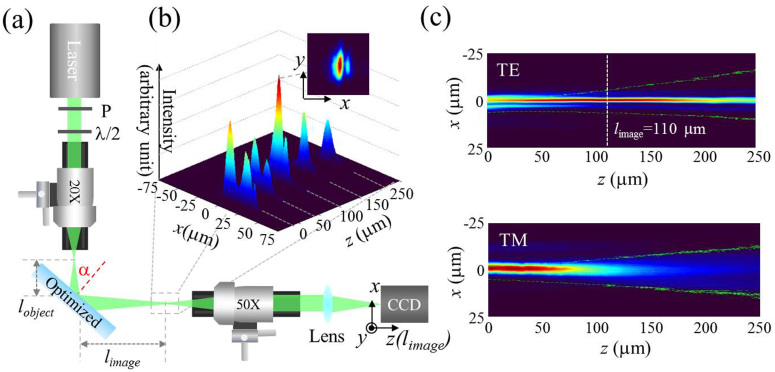
Beam reflections from flat focusing mirror. (a) Measurement scheme, consisting of a CW solid state laser at 532 nm, polarization control, x20 focusing objective and imaging system with x50 objective mounted on a step motor, and f = 20 cm tube lens. (b) Beam profiles along the reflected beam axis direction from 0 to 250 μm for TE polarization, and for α = 46°. Inset shows the beam cross-section at the focal plane. (c) Beam profiles at the optimum incidence angle α = 46° for TE and TM polarization.

**Figure 4:**
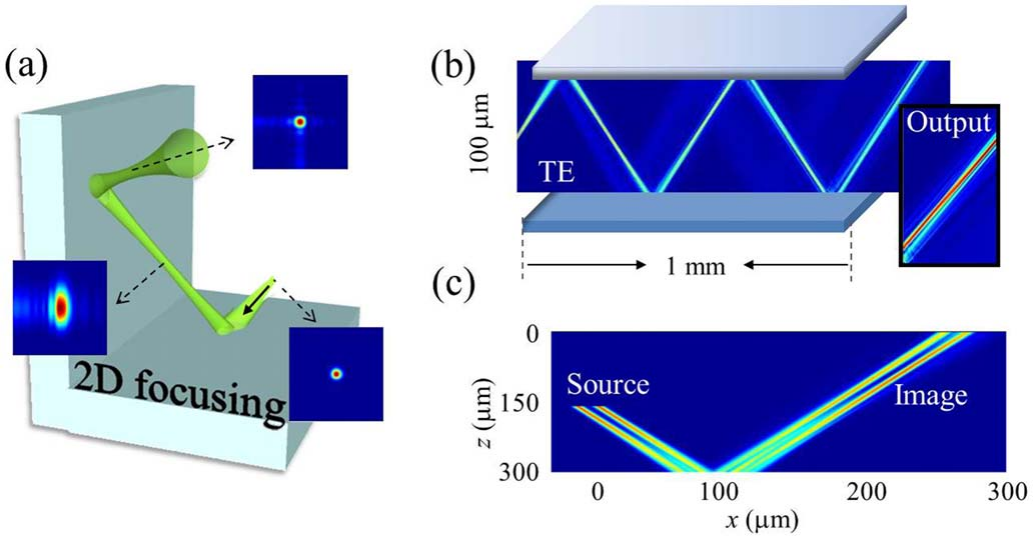
Perspectives of application: (a) 2D focusing from a 3D mirror configuration. (b) Beam propagation inside a Fabry-Perot resonator built from two flat focusing mirrors. (c) Imaging of a light pattern composed of two separated Gaussian beams. The results in this figure are numerical only.

Y.C.C. and K.S. designed the calculations. S.K. conducted the fabrications. Y.C.C. and J.T. were charge of the measurements. K.S. wrote the manuscript and Y.C.C. prepared figures 1–4. M.P., C.C., R.V. R.D. and K.S. recommended the potential applications for the conclusion of the manuscript. All authors reviewed the manuscript.

## Supplementary Material

Supplementary Information video 1

Supplementary Information video 2
