# Supplementary figures and images for: Flat Focusing Mirror
Source: Sci Rep. 2014 Sep 17;4:6326. doi: 10.1038/srep06326 (PMC4165978; doi:10.1038/srep06326)

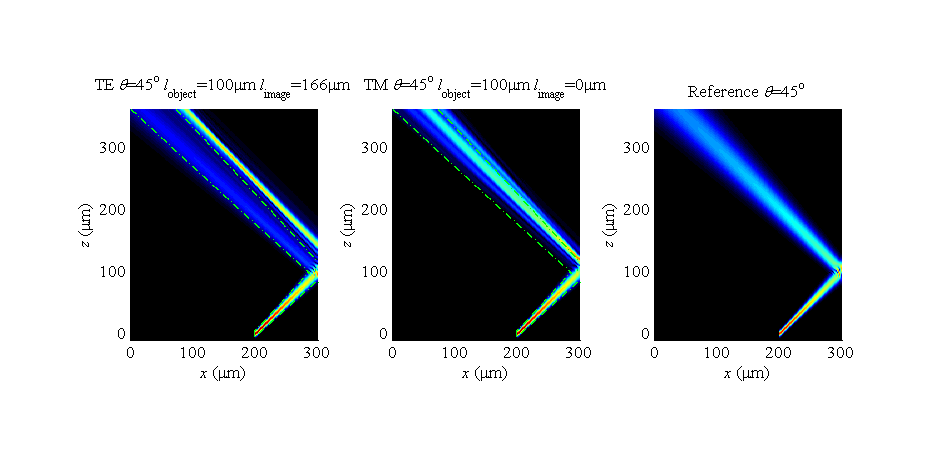

Supplement: Supplementary Information — video 1 [file srep06326-s1.gif]

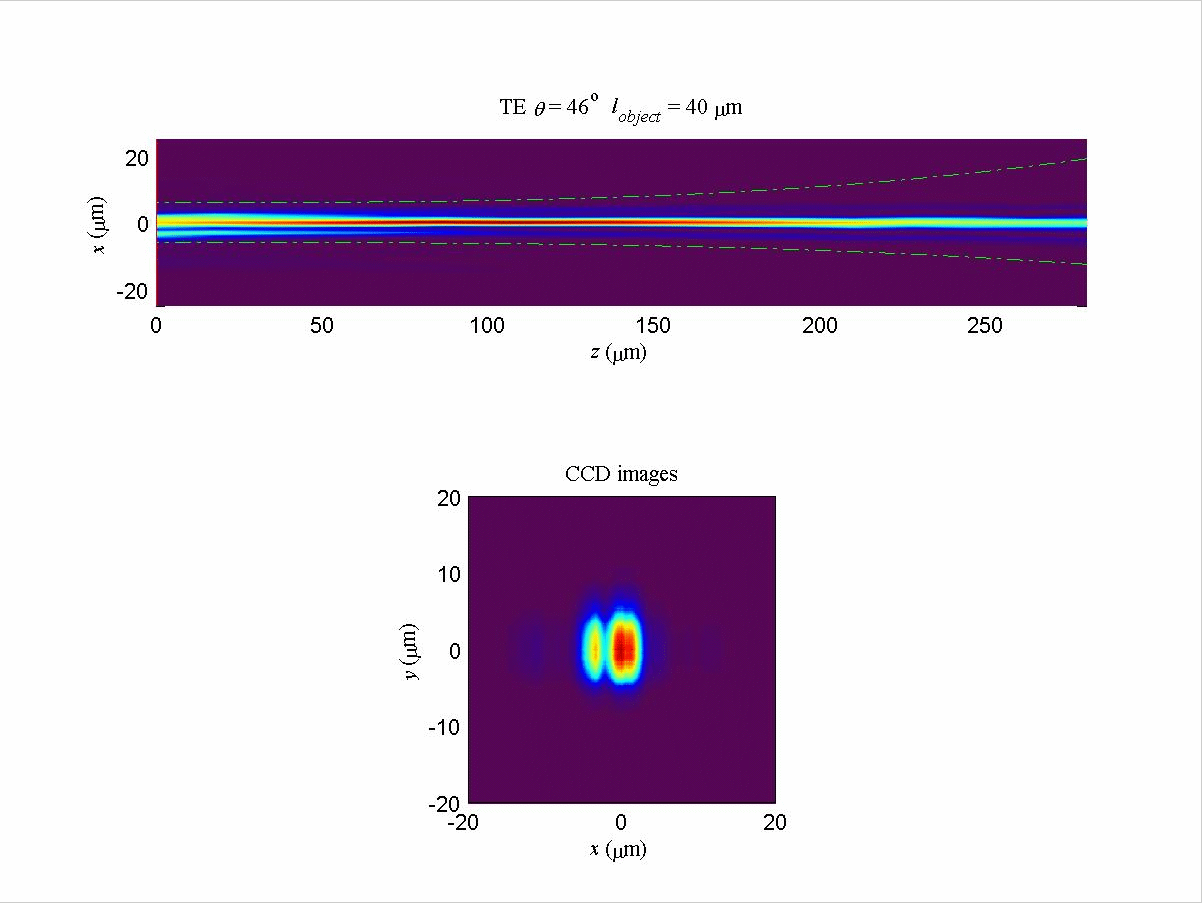

Supplement: Supplementary Information — video 2 [file srep06326-s2.gif]
